# The effect of dietary supplementation with *Clostridium butyricum* on the growth performance, immunity, intestinal microbiota and disease resistance of tilapia (*Oreochromis niloticus*)

**DOI:** 10.1371/journal.pone.0223428

**Published:** 2019-12-09

**Authors:** Hongqin Li, Ying Zhou, Huayun Ling, Li Luo, Desheng Qi, Lin Feng

**Affiliations:** 1 Department of Animal Nutrition and Feed Science, College of Animal Science and Technology, Huazhong Agricultural University, Wuhan, Hubei, China; 2 Animal Feed Science Research Institute, New Hope Liuhe Co., Ltd, Chengdu, Sichuan, China; 3 Technology Center, Sun HY Bio Co., Ltd, Wuhan, Hubei, China; 4 Department of Aquaculture, Southwest University, Chongqing, China; 5 Animal Nutrition Institute, Sichuan Agricultural University, Chengdu, China; USDA-Agricultural Research Service, UNITED STATES

## Abstract

This study was conducted to assess the effects of dietary *Clostridium butyricum* on the growth, immunity, intestinal microbiota and disease resistance of tilapia (*Oreochromis niloticus*). Three hundreds of tilapia (56.21 ± 0.81 g) were divided into 5 groups and fed a diet supplemented with *C*. *butyricum* at 0, 1 x 10^4^, 1 x 10^5^, 1 x 10^6^ or 1 x 10^7^ CFU g^-1^ diet (denoted as CG, CB1, CB2, CB3 and CB4, respectively) for 56 days. Then 45 fish from each group were intraperitoneally injected with *Streptococcus agalactiae*, and the mortality was recorded for 14 days. The results showed that dietary *C*. *butyricum* significantly improved the specific growth rate (SGR) and feed intake in the CB2 group and decreased the cumulative mortality post-challenge with *S*. *agalactiae* in the CB2, CB3 and CB4 groups. The serum total antioxidant capacity and intestinal interleukin receptor-associated kinase-4 gene expression were significantly increased, and serum malondialdehyde content and diamine oxidase activity were significantly decreased in the CB1, CB2, CB3 and CB4 groups. Serum complement 3 and complement 4 concentrations and intestinal gene expression of tumour necrosis factor α, interleukin 8, and myeloid differentiation factor 88 were significantly higher in the CB2, CB3 and CB4 groups. Intestinal toll-like receptor 2 gene expression was significantly upregulated in the CB3 and CB4 groups. Dietary *C*. *butyricum* increased the diversity of the intestinal microbiota and the relative abundance of beneficial bacteria (such as *Bacillus*), and decreased the relative abundance of opportunistic pathogenic bacteria (such as *Aeromonas*) in the CB2 group. These results revealed that dietary *C*. *butyricum* at a suitable dose enhanced growth performance, elevated humoral and intestinal immunity, regulated the intestinal microbial components, and improved disease resistance in tilapia. The optimal dose was 1 x 10^5^ CFU g^-1^ diet.

## Introduction

In recent years, fish has increased in importance as a food source for humans, and aquaculture has rapidly developed [1]. With the expansion of the fish aquaculture industry, fish diseases have increased, resulting in serious economic losses. Antibiotics have been extensively used to prevent and control bacterial diseases [2]. However, the extensive usage of antibiotics may cause some negative effects, such as the emergence of antibiotic-resistant bacteria and antibiotic residues, which may affect the sustainable development of aquaculture and human health [3]. As an alternative, probiotics have received considerable attention in recent years [4]. Studies have shown that probiotics can improve growth [5–10], enhance the immune reponse [11–13], and improve disease resistance in fish [5,6,8–10,13,14]. *Clostridium butyricum*, a typical butyric acid bacterium, is a probiotic that occurs in the intestine of healthy humans [15] and other animals [16] and has been used in a wide range of human and veterinary intestinal diseases [17]. In aquatic animals, studies showed that survival post-challenge with pathogenic bacteria was significantly increased in Chinese drum (*Miichthys miiuy*) [18] and Pacific white shrimp (*Litopenaeus vannamai*) [19,20] fed a diet supplemented with *C*. *butyricum* for 30 and 42 days, respectively. Short-term supplementation with *C*. *butyricum* also improved the disease resistance of rainbow trout [21] and gibel carp [22]. Tilapia is the second most farmed fish worldwide [23]. To our knowledge, there are few studies about the effect of dietary *C*. *butyricum* on tilapia.

Fish disease resistance has been found to be associated with immunity [24,25]. The intestine is an important immune organ [25]. The intestinal immune response is related to inflammation mediated by cytokines [26]. Studies have shown that probiotics upregulate the gene expression of interleukin 8 (*IL-8*) in the intestine of rainbow trout (*Oncorhynchus mykiss*) [27] and grouper *(Epinephelus coioides*) [28] and that of tumour necrosis factor α (*TNF-a*) in the of intestine hybrid tilapia (*Oreochromis niloticus* x *Oreochromis aureus*) [29]. However, no studies have examined the effect of *C*. *butyricum* on cytokines in fish intestine. In HT-29 human colonic epithelial cells, *C*. *butyricum* improved the gene expression of the pro-inflammatory cytokines *TNF-α* and *IL-8* [17,30] and the anti-inflammatory cytokine *-IL-10* [17,31]. *C*. *butyricum* improved *IL-6* and *IL-8* gene expression in interstitial cells of Cajal (ICCs) [32]. Toll-like receptor (*TLR*) signalling pathways play an important role in the recognition of probiotics and activation of the intestinal immune system in mammals [28]. *C*. *butyricum* induced macrophages in inflamed mucosa producing IL-10 to prevent acute experimental colitis via the *TLR2*/Myeloid differentiation factor 88 (*MyD88*) signalling pathway [33]. *TLR2* silencing alleviated *C*. *butyricum*-induced *IL-6* and *IL-8* expression and significantly inhibited *C*. *butyricum*-induced cell viability in ICCs [32]. *C*. *butyricum* upregulated *TLR2* gene expression in HT-29 human colonic epithelial cells [17] and upregulated the gene expression of *TLR2* and *MyD88* in the intestine of weaning rex rabbits [34]. However, the roles of fish TLRs in the C. butyricum-induced intestinal immune response have not been reported. In grouper (*Epinephelus coioides*), the probiotic *Psychrobacter sp*. SE6 upregulated the gene expression of *TLR2* and *MyD88* in the intestine [28], which suggests that *TLR2* signalling may play a key role in the modulation of intestinal immunity by *C*. *butyricum*.

The intestinal structural integrity is the foundation of intestinal immunity in fish [35]. The integrity of intestinal mucosa cells is reflected in serum diamine oxidase (DAO) activity [36]. Tight junction proteins are critical for maintaining the intercellular structural integrity of the intestine in mice [37,38]. A previous study found that dietary supplementation with *C*. *butyricum* decreased serum DAO in broiler chickens [39] and piglets [40]. Dietary *C*. *butyricum* upregulated the protein expression levels of *ZO-1*, *claudin-3*, and *occludin* in the intestines of weaned piglets [40] and the gene expression levels of *ZO-1* and *occludin* in the ileum and colon of weaning rex rabbits [34]. To date, no reports have addressed the effect of *C*. *butyricum* on DAO gactivity and tight junction proteins in fish. Intestinal tissue showed close intestinal epithelium connections and healthy morphology in kuruma shrimp (*Marsupenaeus japonicas*) [41] and Pacific white shrimp (*Litopenaeus vannamei*) [42] fed diets supplemented with *C*. *butyricum*, suggesting that *C*. *butyricum* might affect intestinal structural integrity in fish, which needs to be further studied.

The intestinal microbiome is part of the host and interacts with the host immune system [43]. Studies revealed some disease association with dysbiosis or abnormal composition of the intestinal microbiome [44]. Studies have shown that *C*. *butyricum* can modify the intestinal microbiota. In vitro, *C*. *butyricum* inhibited the growth and adherence of potential pathogens to fish intestinal epithelial cells [45,46]. The potential intestinal pathogens were decreased, and the beneficial bacteria were increased in response to dietary *C*. *butyricum* in *Litopenaeus vannamei* [47], broiler chickens [48,49] and laying hens [50], and mice [16]. These data suggest that *C*. *butyricum* might affect the intestinal microbiome in fish, which needs to be further studied.

Therefore, the aim of the present study was to evaluate the effects of dietary supplementation with *C*. *butyricum* on growth performance and disease resistance in tilapia and the underlying mechanisms involved.

## Materials and methods

### Bacterial strains

*C*. *butyricum* (China Center for Type Culture Collection accession NO. M2014537) used in this experiment was obtained from Sun HY Bio Co., Ltd., Wuhan, China. *Streptococcus agalactiae* was kindly provided by Guangdong Ocean University, Zhanjiang, China.

### Diets and feeding management

*C*. *butyricum* was cultured with reinforced Clostridium medium (RCM) (tryptone 10 g, beef extract 10 g, yeast extract 3 g, glucose 5 g, soluble starch 1 g, sodium chloride 5 g, sodium acetate 3 g, L-cysteine hydrochloride 0.5 g, distilled water 1000 mL) at 37°C for 24 h under anaerobic conditions. After cultivation, the bacteria were collected by centrifugation and resuspended in sterile saline at a concentration of 1x10^9^CFU g^-1^. The bacterial cell quantity was examined using the spread RCM plate count method. The formulation of the basal diet is presented in Table 1. The bacterial suspension was gently sprayed on the basal diet, followed by thorough mixing, at five concentrations: 0 (control group), 1 x 10^4^, 1 x 10^5^, 1 x 10^6^ and 1 x 10^7^ CFU g^-1^ diet (denoted as CG, CB1, CB2, CB3 and CB4, respectively). The experimental diets were pelletized and stored at -20°C until use [51,52].

**Table 1:** Composition and nutrient content of the basal diet.

Ingredients	g kg^-1^ diet	Nutrients content^a^	g kg^-1^ diet
**Fish meal**	50	Crude protein	323.4
**Soy bean meal**	300	Crude fat	60.6
**Cottonseed meal**	130	Crude fibre	48.3
**Rapeseed meal**	160	Crude ash	47.6
**Wheat flour**	200		
**Rice bran**	54		
**Cassava starch**	40		
**Soybean oil**	37		
**Ca(H**_**2**_**PO**_**4**_**)**_**2**_	20		
**Choline chloride (50%)**	3		
**Vitamin premix**^**b**^	1		
**Mineral premix**^**b**^	5		

^a^ The crude protein, crude fat, crude fibre and crude ash content are measured values.

^b^ Kindly provided by New Hope Liuhe Co., Ltd, Chengdu, China.

The experimental protocol was approved by the Animal Care Advisory Committee of Huazhong Agricultural University. Juvenile tilapias were obtained from the Far East of Guangxi Agriculture and Animal Husbandry Fishery Development Co., Ltd. (Guangxi, China). Prior to the experiment, tilapia were cultured in experimental conditions (50 L/tank and 30 fish/tank) for 14 d and fed with the basal diet. After adaptation to the farming system, a total of 300 healthy tilapias (initial average weight 56.16 ± 0.82 g) were randomly divided into 15 tanks (50 L) with 20 fish/tank and fed one of five experimental diets to apparent satiety four times daily for 56 days. During the experiment, the water temperature and pH were 25–28°C and 7.0–7.5, respectively, and the dissolved oxygen content remained above 5 mg L^-1.^

### Sample collection

At the end of the feeding trial, 5 fish per tank were anesthetized using tricaine methane sulfonate (MS222) according to Zhou et al. [53]. Blood samples were collected into non-heparinized tubes from the caudal vein and allowed to clot for 12 h (at 4°C). Then, serum was isolated by centrifugation (5000 g, 5 min) and stored at -20°C until further analysis as described by Hoseinifar et al. [54]. After blood sampling, these fishes were slaughtered and the hindguts were taken out. The luminal contents and hindgut tissues were collected and deposited at −80°C for later analysis. The remaining fishes were used for the challenge test.

### Challenge test

The challenge test was approved by the Animal Care Advisory Committee of Huazhong Agricultural University and was conducted after the feeding trial. The remaining 45 fish in each group (triplicate) were anesthetized with MS222, and injected intraperitoneally with 0.3 mL of PBS containing 1.5 x 10^6^ CFU bacteria mL^-1^. Then, these infected fish were returned to the original tank. All water quality parameters were the same as those in the feeding trial. The tanks were monitored every 2 h by a trained aquaculture technician. According to the previous description [55], any dead fish were removed, and any severely morbid fish were immediately anaesthetized and euthanized once noticed. All dead fish were examined for typical symptoms of streptococcosis. The challenge test lasted for 14 days.

### The analysis of serum and hindgut tissue samples

According to Li et al. [56], serum total antioxidant capacity (T-AOC) and malondialdehyde (MDA) were analysed via kits provided by Nanjing Jiancheng Biological Engineering Research Institute (Nanjing, China). DAO activity was analysed via kits provided by Hangzhou Nuoyang Biological Technology Co. Ltd. (Hangzhou, China) according to Lei et al. [57]. Kits provided by Zhejiang Elikan Biological Technology Co. Ltd. (Wenzhou, China) were used for the determination of C3 and C4 according to Sun et al. [58].

Thirty hindgut tissue samples, six samples from each group, were used for RNA extraction. Total RNA was extracted using TRIzol according to the manufacturer's instructions (Aidlab Biotechnologies, Beijing, China). The quantity and quality of RNA were assessed using an ultraviolet spectrophotometer (Shanghai Sunny Hengping, Shanghai, China); the ratios of absorbance at 260 and 280 nm (A260/A280) were used to assess RNA purity and were between 1.8 and 2.0. Then, cDNA was synthesized using the Prime Script^™^ RT Reagent Kit according to the manufacturer's protocol (TaKaRa, Tokyo, Japan). Real-time PCR was applied to evaluate gene expression levels using gene-specific primers as shown in Table 2. β-actin was used as the house-keeping gene to standardize the data. Real-time PCR assays were conducted in an ABI 7900 real-time PCR detection system (Applied Biosystems, California, USA) with 20 μL reaction volumes containing: 4 μL of cDNA (10 times dilution), 0.4 μL of forward primer (100 μM), 0.4 μL of reverse primer (100 μM), 10 μL of 2× SYBR Premix Ex Taq^TM^ (TaKaRa, Tokyo, Japan) and 5.2 μL of deionized H_2_O. The cycling conditions were as follows: 50°C for 2 min, 95°C for 10 min and then 40 cycles at 95°C for 30 s and 60°C for 30 s. The relative gene expression levels were analysed using the 2^-ΔΔCT^ method according to Livak and Schmittgen [59].

**Table 2:** The gene specific primers sequences.

Gene	Primer	Sequence	NCBI Gene ID
***β-actin***	Forward	5’- TCCACGAAACCACCTACAACA -3’	100534414
	Reverse	5’- CCAGACGGAGTATTTACGCTCA -3’	
***claudin* 1**	Forward	5‘- CTTCACTCTGGTCGCCGTGTC -3’	100705074
	Reverse	5‘- GCAGCAAAGCATAGATCCTCCC -3	
***occludin***	Forward	5‘- AATCGGGATAATCTCCTACA -3’	100695261
	Reverse	5‘- TTGGTCCTCTTTGCTATTTG -3’	
***IL10***	Forward	5‘- AGATGTCACCCAGTGTAGGAA -3’	100694754
	Reverse	5‘- AAGCCAGGTACGTCTCAAAGT -3	
***IL-8***	Forward	5‘- ACCTGTGAAGGCATGGGTGT -3’	100534479
	Reverse	5‘- TCGCAGTGGGAGTTGGGAAG -3	
***TNF-a***	Forward	5‘- TCGTCGTCGTGGCTCTTTGT -3’	100534578
	Reverse	5‘- GCCTTGGCTTTGCTGCTGAT -3	
***TLR*2**	Forward	5‘- CATTCTGCTATCTGTGGTGCTGT -3’	100694547
	Reverse	5‘- GCTGCTTTCGCTTGGCTCCTCTA -3	
***MyD*88**	Forward	5‘- ATGCCTTCATCTGCTACTGC -3’	100700534
	Reverse	5‘- ATCCGTTTACACCTCTTCTCG -3	
***IRAK*-4**	Forward	5‘- ATACAAAGGTCTCCTGGATGA -3’	100707440
	Reverse	5‘- AGCAAGCCAGTCGGTCTAAT -3	

### The analysis of the intestinal microbiota

According to the results of the feeding trial and challenge test, the most effective treatment in the present study was considered to be the CB2 group. Therefore, a comparison of bacterial structure and composition of the intestine between the CG and CB2 groups was performed. Twelve hindgut content samples, six each from the CG and CB2 groups, were used for analysis of the intestinal microbiota. Total microbial DNA was extracted using a TIANamp Stool DNA Kit (TIANGEN, Beijing, China) according to the manufacturer's guidelines, and the quality of the DNA samples was determined by agarose electrophoresis and a Nanodrop 8000 spectrophotometer (Thermo Fisher Scientific, Brisbane, Australia). The V3-V4 hypervariable region of the bacterial 16S rRNA gene was amplified by PCR (95°C for 2 min, followed by 25 cycles at 95°C for 30s, 55°C for 30 s, and 72°C for 30 s and a final extension at 72°C for 5 min) using forward primer 338F (5’- ACTCCTACGGGAGGCAGCAG-3’) and reverse primer 806R (5’–GGACTACHVGGGTWTCAAT-3’). PCR reactions were conducted in triplicate using a 20 μL reaction mixture consisting of template DNA (10 ng), FastPfu Polymerase (0.4 μL), each primer at 5 μM (0.8 μL), 2.5 mM dNTPs (2 μL) and 5 ×FastPfu Buffer (4 μL). Agarose gels (2%) were used to extract the amplicons, which were purified using the AxyPrep DNA Gel Extraction Kit (Axygen Biosciences, Union City, CA, U.S.) according to the manufacturer’s instructions and quantified using QuantiFluor^™^ -ST (Promega, U.S.). The amplicons from each sample were pooled in equimolar amounts and sent to Majorbio Bio-Pharm Technology Co., Ltd (Shanghai, China). The Illumina MiSeq platform was used to perform paired-end sequencing (2 × 250/300 bp) of these amplicons according to standard protocols.

In the present study, the Illumina sequencing reads were mainly analysed using QIIME pipeline software (version 1.9.1). The poor/low quality sequences, including those with uncertain nucleotides and three continuous nucleotides with an average quality of less than 20 over a 50bp sliding window, were removed. Operational Taxonomic Units (OTUs) were clustered with 97% similarity cut off using UPARSE (version 7.1 http://drive5.com/uparse/), and chimeric sequences were identified and removed using UCHIME. The taxonomy of each 16S rRNA gene sequence was analysed by RDP Classifier (http://rdp.cme.msu.edu/) against the Silva (SSU123) 16S rRNA database using a confidence threshold of 0.7. Mothur was also used to calculate the alpha diversity (Shannon index, Chao1, ACE and Sobs) and rarefaction curves. R programming language was used to perform the Venn diagram analysis. Afterwards, all sequence data were deposited to the SRA of the NCBI database under BioProject PRJNA559959.

### Calculations and statistical analysis

Specific growth rate (SGR), feed conversion ratio (FCR) and cumulative mortality were calculated for each treatment according to [60,61].

SGR = {[ln (mean final weight) − ln (mean initial weight)] /number of days} x 100

FCR = feed intake (g)/wet weight gain (g)

Cumulative mortality = 100 x the number of dead fish/15

A nonparametric test of two samples was performed to assess the differences in bacterial relative abundance and diversity indices between the CB2 and CG groups. One-way ANOVA and Duncan's multiple comparison test were performed to assess the differences in growth performance, cumulative mortality, serum complement concentration, antioxidant capacity, gene expression, and DAO activity among the five groups. The significance level was set at *P* < 0.05. Statistical analyses were conducted using SPSS Statistics software v. 19.0 (IBM, Armonk, NY, USA). Results are presented as means ± standard deviation (SD).

## Results

### Growth performance

The effects of dietary *C*. *butyricum* on SGR, feed intake and FCR of tilapia are presented in Table 3. No fish died during the feeding experiment. SGR and feed intake were significantly improved in tilapia that were fed a diet with *C*. *butyricum* at a level of 1 x 10^5^ CFU g^-1^ diet (*P* < 0.05), but FCR was not affected by dietary *C*. *butyricum* supplementation (*P* > 0.05).

**Table 3:** Growth Performance of tilapia fed diets supplemented with *C*.*butyricum*.

	CG	CB1	CB2	CB3	CB4
**Initial body weight (g)**	55.93±1.25^a^	56.53±0.81^a^	56.00±0.57^a^	56.40±0.52^a^	55.90±1.10^a^
**Final body weight (g)**	178.77±4.70^a^	187.20±8.55^ab^	197.25±7.42^b^	189.33±9.04^ab^	184.53±7.22^ab^
**SGR**	2.00±0.01^a^	2.06±0.10^ab^	2.17±0.08^b^	2.09±0.10^ab^	2.06±0.04^ab^
**Feed intake (g fish**^**-1**^**)**	145.68±8.27^a^	156.91±6.65^ab^	163.24±6.39^b^	156.94±7.47^ab^	156.09±3.74^ab^
**FCR**	1.19±0.04^a^	1.20±0.04^a^	1.16±0.02^a^	1.18±0.03^a^	1.22±0.03^a^

SGR: Specific growth rate. FCR: feed conversion ratio.

Values are means ± standard deviation of three replicate groups. Mean values with the different superscripts in the same row are significantly different (P < 0.05).

### Cumulative mortality

**Fig 1:**
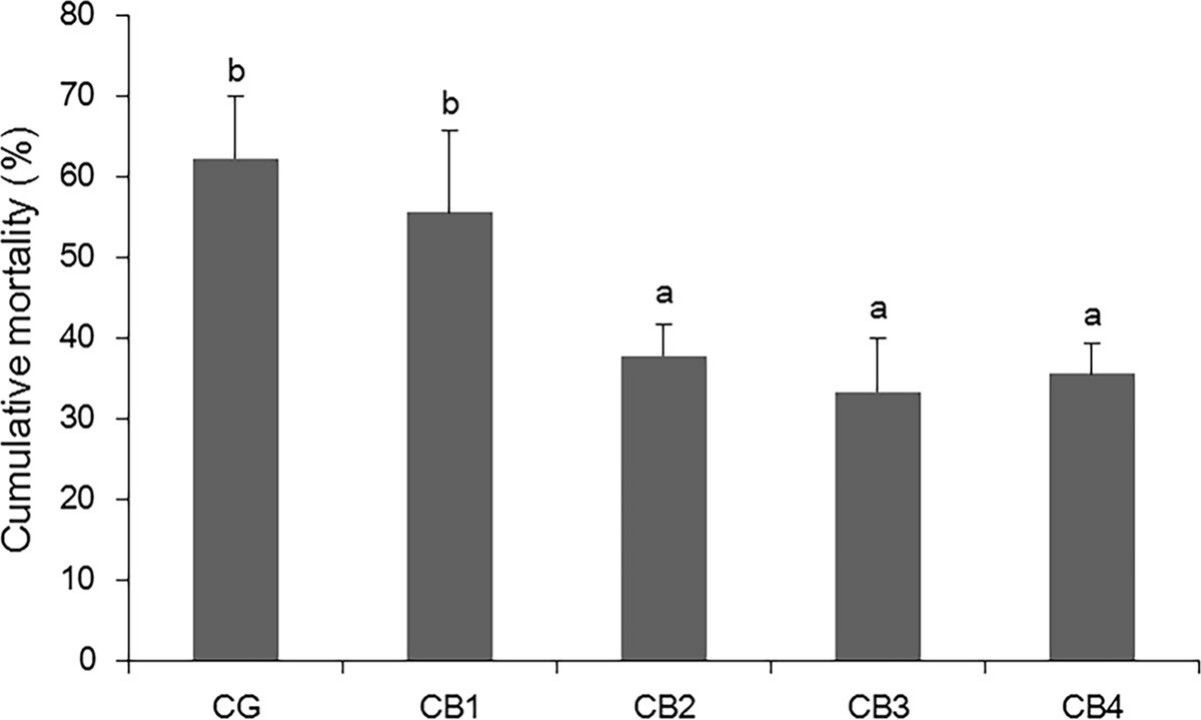
The Effects of dietary *C*. *butyricum* on the cumulative mortality of tilapia post- challenge with *S*. *agalactiae*.

The cumulative mortality of fish at 14 days post-challenge with *S*. *agalactiae* is presented in Fig 1. *C*. *butyricum* significantly decreased the cumulative mortality of tilapia at levels of 1 x 10^5^, 1 x 10^6^ and 1 x 10^7^ CFU g^-1^ diet (*P* < 0.05). Dead fish showed the typical symptoms of streptococcosis such as darkness of the body surface, exophthalmos, and basal fin haemorrhage.

### Serum complement concentration

**Fig 2:**
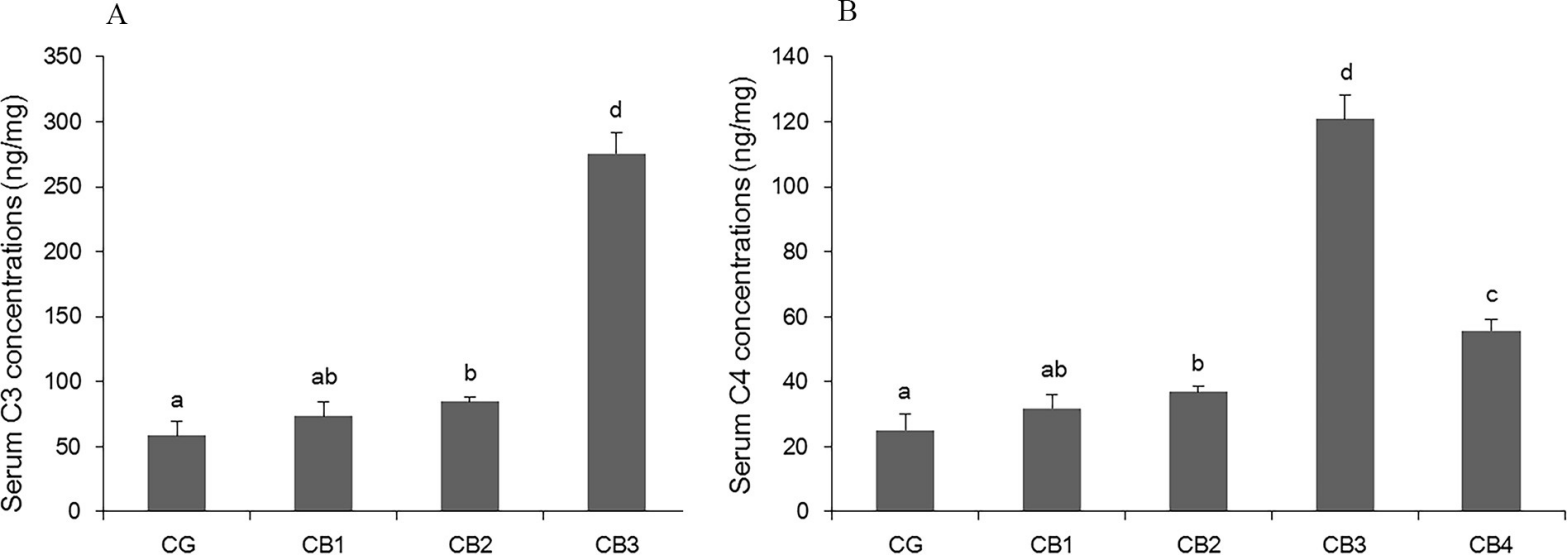
The effects of dietary *C*. *butyricum* on serum complement 3 (C3) and 4 (C4) concentrations. A: The effects on serum C3; B: The effects on serum C4.

The effects of dietary *C*. *butyricum* on serum C3 and C4 concentrations of tilapia are presented in Fig 2. Serum C3 and C4 concentrations increased significantly with dietary *C*. *butyricum* at levels of 1 x 10^5^, 1 x 10^6^ and 1 x 10^7^ CFU g^-1^ diet (*P* < 0.05), with the highest levels observed in the CB3 group receiving the diet supplemented with *C*. *butyricum* at 1 x 10^6^ CFU g^-1^ diet (*P* < 0.05).

### Antioxidant capacity

**Fig 3:**
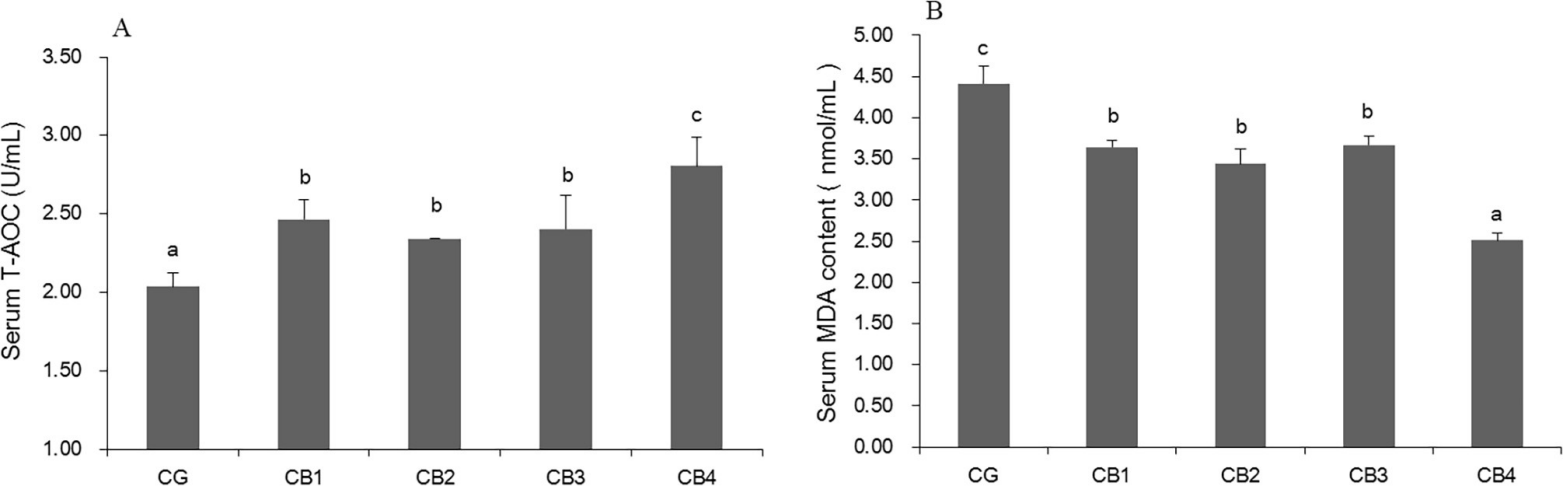
The effects of dietary *C*. *butyricum* on the serum T-AOC, MDA contents. A: The effects on serum T-AOC; B: The effects on serum MDA.

The effects of dietary *C*. *butyricum* on the serum T-AOC and MDA contents of tilapia are presented in Fig 3. Serum T-AOC increased significantly with dietary *C*. *butyricum*, supplementation, with the highest levels observed in the CB4 group receiving the diet supplemented with *C*. *butyricum* at 1 x 10^7^ CFU g^-1^ diet (*P* < 0.05). Serum MDA content significantly decreased with dietary *C*. *butyricum* supplementation, with the lowest content observed in the CB4 group receiving *C*. *butyricum* at 1 x 10^7^ CFU g^-1^ diet (*P* < 0.05).

### Gene expression of cytokines, *TLR2*, *MyD88*, and *IRAK-4*

**Fig 4:**
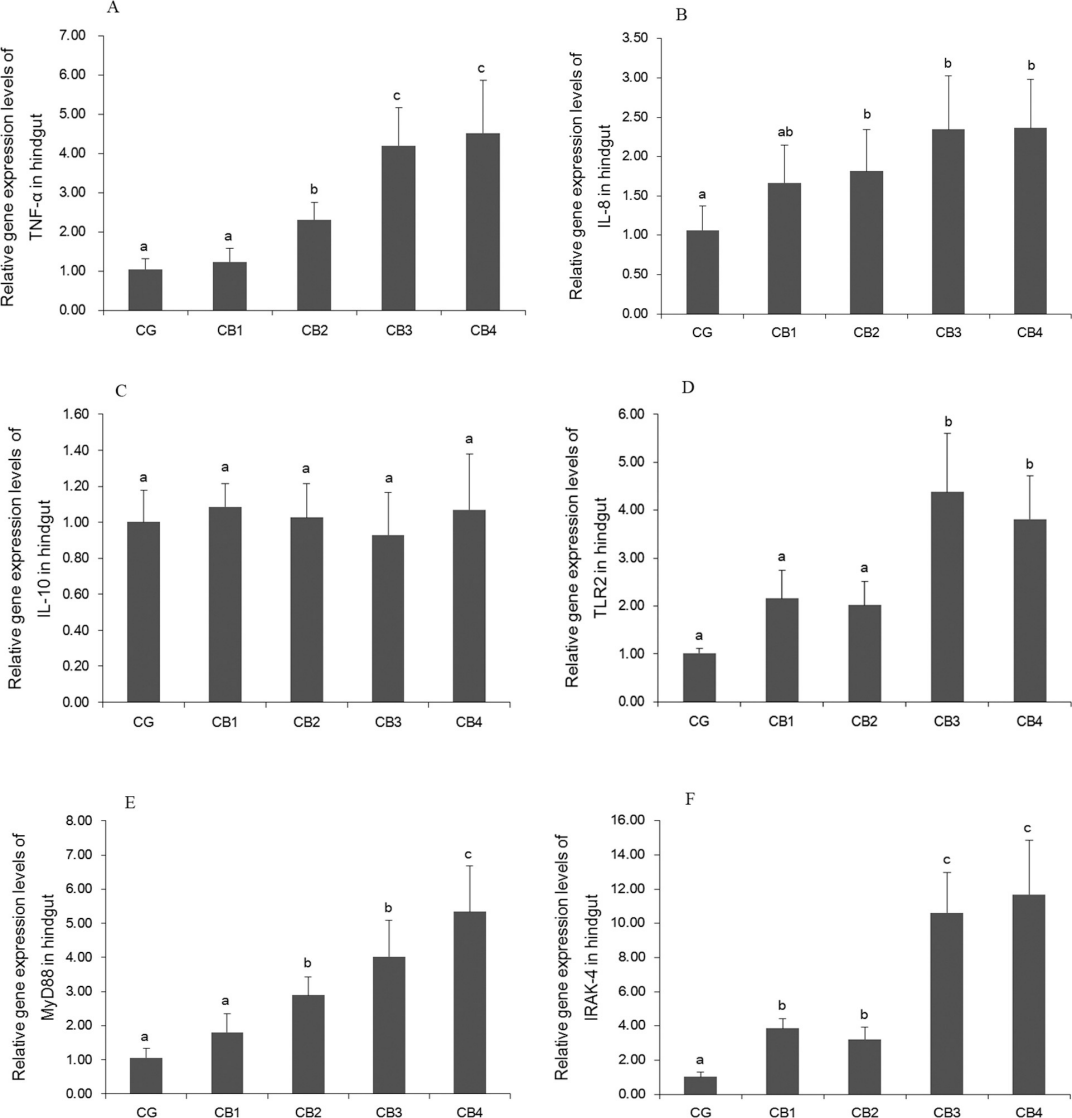
The gene expression of *TNF-α*, *IL-8*, *IL-10*, *TLR2*, *MyD88*, *IRAK-4* in the Hindgut of tilapia. A: The effects on the gene expression levels of *TNF-α*; B: The effects on the gene expression levels of *IL-8*; C: The effects on the gene expression levels of *IL-10*; D: The effects on the gene expression levels of *TLR2;* E: The effects on the gene expression levels of *MyD88*; F: The effects on the gene expression levels of *IRAK-4*.

The effects of dietary *C*. *butyricum* on the gene expression of cytokines, *TLR2*, *MyD88* and *IRAK-4* in the hindgut of tilapia are presented in Fig 4. Compared with the unsupplemented group, gene expression of *TNF-α*, *IL-8* and *MyD88* was significantly upregulated by *C*. *butyricum* at levels of 1 x 10^5^, 1 x 10^6^ and 1 x 10^7^ CFU g^-1^ diet (*P* < 0.05). *TLR2* gene expression was significantly upregulated by *C*. *butyricum* supplementation, and significant upregulation was observed at levels of 1 x 10^6^ and 1 x 10^7^ CFU g^-1^ diet relative to the control group (*P* < 0.05). *C*. *butyricum* significantly upregulated *IRAK-4* gene expression (*P* < 0.05) but did not affect *IL-10* gene expression (*P* > 0.05).

### The intestinal physical barrier

**Fig 5:**
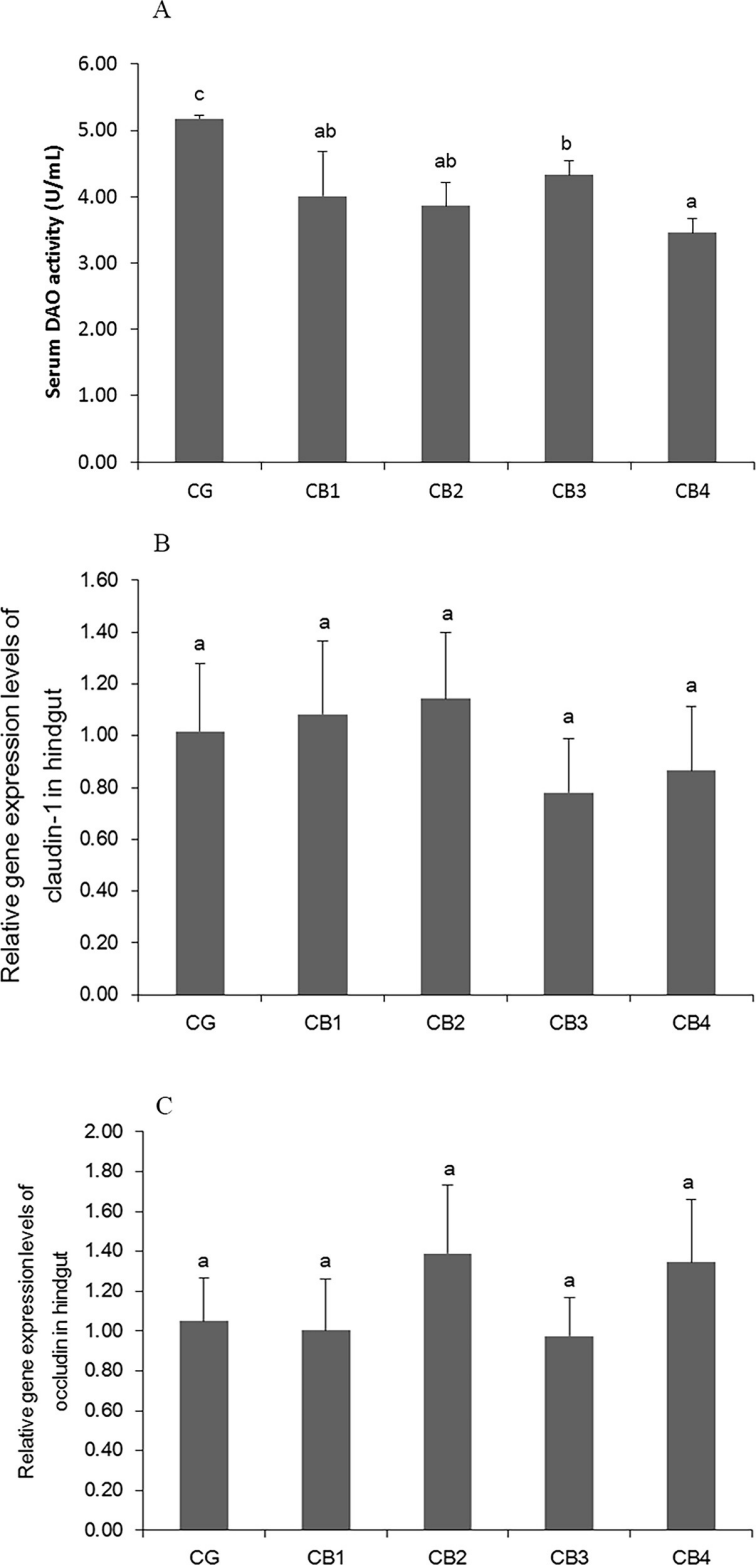
The serum DAO activity and gene expression of *Claudin -1* and *Occludin* in the hindgut. A: The effects on serum DAO activity; B: The effects on the gene expression levels of claudin -1; C: The effects on the gene expression levels of occudin.

The effects of dietary *C*. *butyricum* on serum DAO activity and the relative gene expression of *claudin-1* and *occludin* in the hindgut of tilapia are presented in Fig 5. Serum DAO activity significantly decreased with dietary *C*. *butyricum* supplementation and was lowest in the CB4 group receiving *C*. *butyricum* at 1 x 10^7^ CFU g^-1^ diet (*P* < 0.05). The relative gene expression of *claudin-1* and *occludin* was not significantly affected by *C*. *butyricum* supplementation (*P* > 0.05).

### Intestinal microbiota analyses

**Fig 6:**
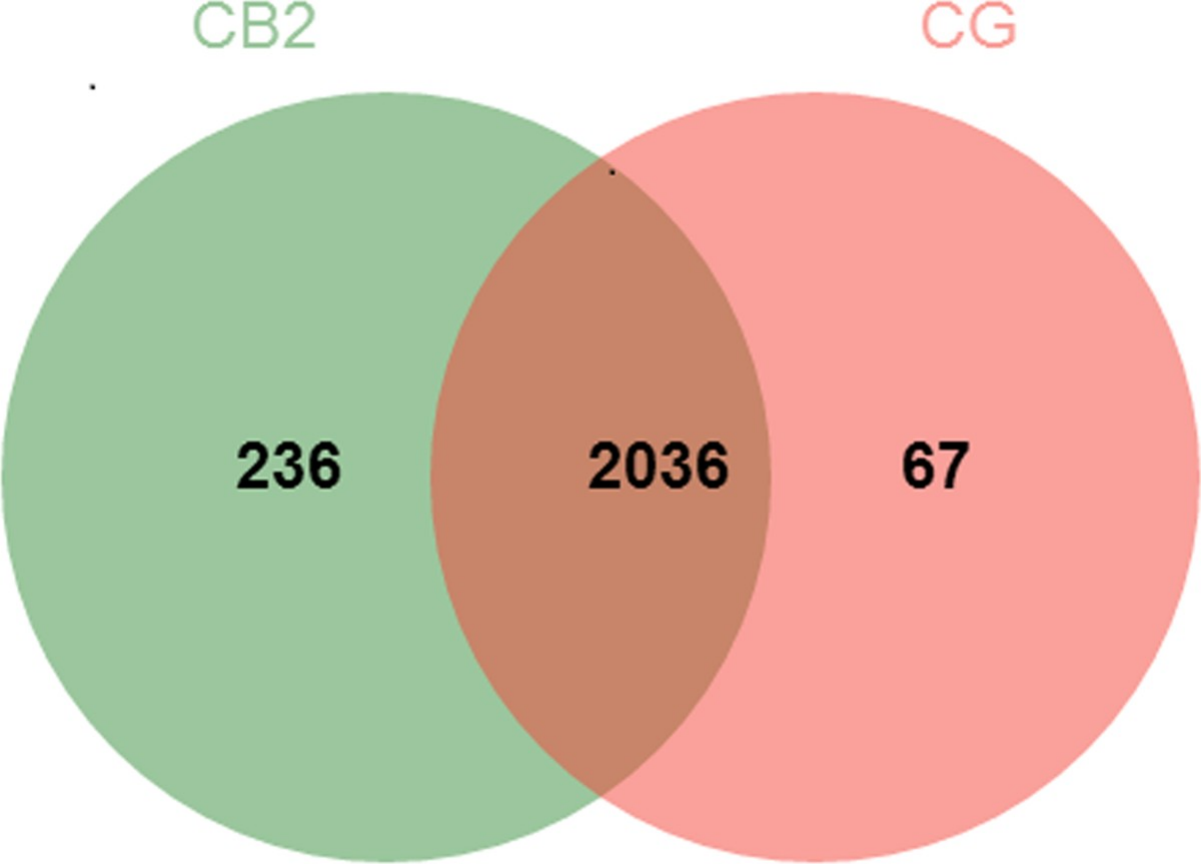
Venn plot showing the shared and unique OTUs.

A total of 436184 valid sequences were obtained through Illumina sequencing analysis in the present study, with an average of 36348 (SD = 3585) per sample. A total of 2339 OTUs were identified at the 97% similarity level. There were 2036 OTUs shared between CG and CB2, 236 unique OTUS for the CB2 group and 67 unique OTUS for the CG (Fig 6).

**Fig 7:**
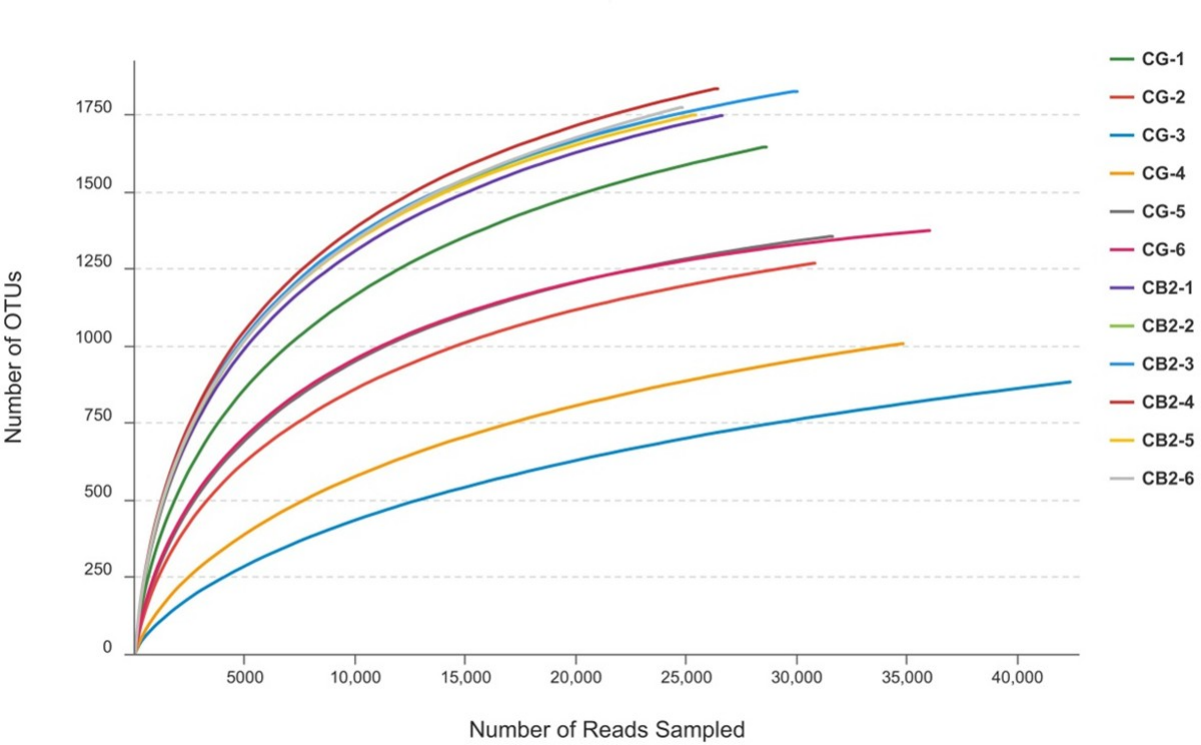
Rarefaction curves of samples.

A rarefaction test was performed at the OTU level, and the results are presented in Fig 7. The rarefaction curves tended to approach the saturation plateau, indicating that the majority of microorganisms were revealed in the present study. Alpha diversity indices are presented in Table 4. Compared with the CG group, Sobs, Shannon, Ace and Chao1 indices were significantly increased by dietary supplementation with *C*. *butyricum* (1 x 10^5^ CFU g^-1^ diet) (*P* < 0.05).

**Table 4:** Comparison of the diversity indices of bacteria communities between the CG and CB2 groups.

Group	Sobs	Ace	Chao1	Shannon
**CG**	446.67±50.27^a^	489.78±47.28^a^	490.30±44.39^a^	3.32±0.84^a^
**CB2**	514.00±7.90^b^	547.30±5.30^b^	552.24±5.73^b^	4.99±0.05^b^

Sobs: the observed richness; Ace: abundance-based coverage estimator; Vertical bars represented the means ± standard deviation (N = 6). Data indicated with different letters were significantly different (P < 0.05) between CG and CB2 group.

**Fig 8:**
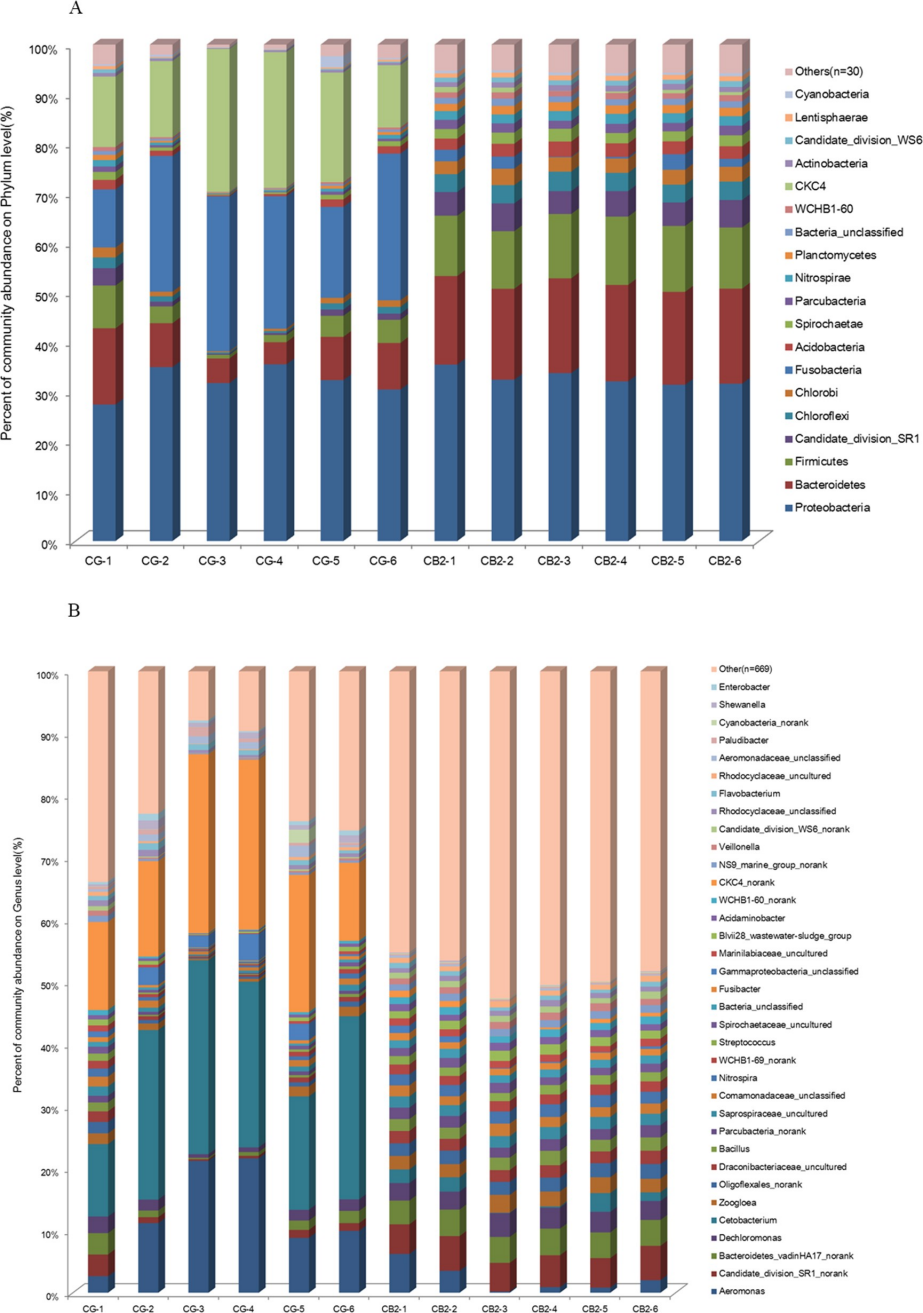
Bacterial composition at the phylum and genus levels of the CB2 and CG groups. A: The bacterial compositions at the phylum level. B: The bacterial compositions at the genus level. Only the phylum and genus whose relative abundance was more than 1% were presented.

**Fig 9:**
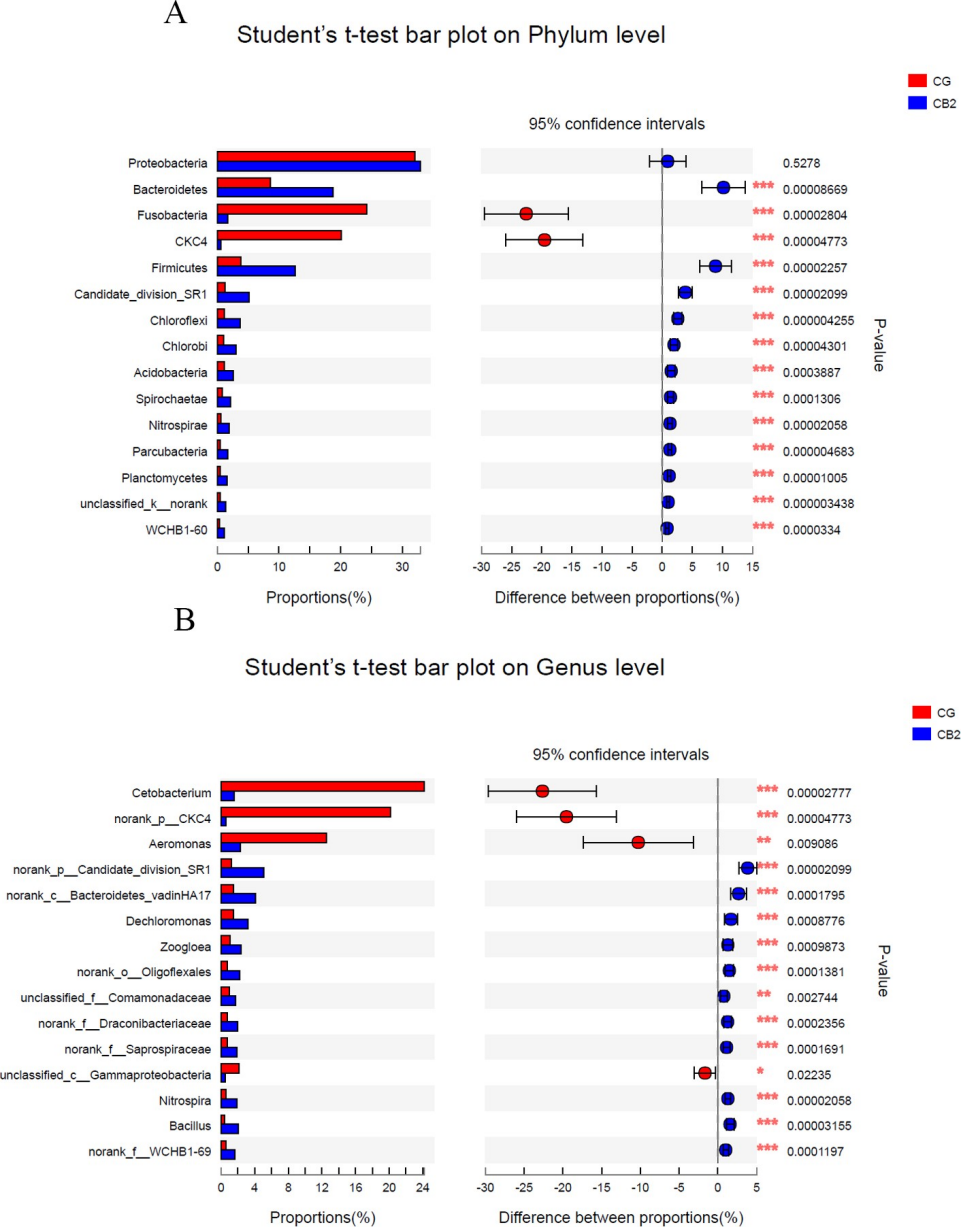
The differences at the phylum and genus level between the CB2 and CG groups. A: at the phylum level. B: at the genus level. The vertical axis represented the species name at the phylum or genus level. Each column corresponding to the species represented the average relative abundance of the species in the CG and CB2 groups. Different colors represented different groups. Only the top15 most abundant genera were presented. 0.01< *P* < = 0.05 *, 0.001< *P* < = 0.01 **, *P* < = 0.001 ***.

A total of 49 phyla, 108 classes, 210 orders, 394 families and 704 genera were identified in the present study. The bacterial compositions at the phylum and genus levels are presented in Fig 8. There were 42 phyla whose relative abundances were significantly different between the CG and CB2 groups, and the top 15 most abundance phyla are presented in Fig 9A. Compared to the control group, the relative abundances of Bacteroidetes, Firmicutes, Candidate-division-SR1, Chloroflexi, Chlorobi, Acidobacteria, Spirochaetae, Nitrospirae, Parcubacteria, Planctomycetes and WCHB1-60 were significantly enriched (*P* < 0.05), and those of Fusobacteria and CKC4 were significantly weakened in the CB2 group (*P* < 0.05). Proteobacteria was statistically the same between the two groups (*P* > 0.05). There were 354 genera whose relative abundances were significantly different between the CG and CG2 groups (*P* < 0.05), and the top15 most abundant genera are presented in Fig 9B. Compared with the CG group, the relative abundances of *Cetobacterium*, CKC4, *Aeromonas* and *Gammaproteobacteria* were significantly lower (*P* < 0.05), and those of Candidate_division_SR1_norank, Bacteroidetes_vadinHA17_norank, *Dechloromonas*, *Zoogloea*, *Oligoflexales_nora*n, *Comamonadaceae*_unclassified, *Draconibacteriaceae*_uncultured, *Saprospiraceae*_uncultured, *Nitrospira*, *Bacillus*, and WCHB1-69_norank were significantly higher in the CB2 group (*P* < 0.05).

## Discussion

Probiotics have been extensively researched, and their effects on the growth and health of aquatic animals have been verified in recent years [3]. Compared with other probiotics, such as *Bacillus* and *Lactobacillus*, *C*. *butyricum* has a stronger tolerance to many antibiotics, higher temperature, and lower pH environment and has a wide range of application prospects [40,62]. Previous studies showed that a suitable dose of dietary *C*. *butyricum* increased growth performance in Pacific white shrimp (*Litopenaeus vannamei*) [19,20,42], black tiger shrimp (*Penaeus monodon*) [63], *Macrobrachium rosenbergii* [64], Chinese drum (*Miichthys miiuy)* [65] and silver pomfret (*Pampus argenteus*) [66]. The present study also found that dietary *C*. *butyricum* at a level of 1 x 10^5^ CFU g^-1^ diet significantly improved the growth performance of tilapia. Fish growth performance is related to disease resistance [67]. Mortality post-challenge is an indicator of disease resistance [68,69]. In the present study, cumulative mortality post-challenge with *S*. *agalactiae* was decreased in tilapia fed a diet supplemented with *C*. *butyricum* at levels of 1 x 10^5^, 1 x 10^6^ and 1 x 10^7^ CFU g^-1^diet. Similar results have been obtained in Chinese drum and rainbow trout. Pan et al. [18] reported that survival post-challenge with *V*. *anguillarum* or *A*. *hydrophila* was enhanced in Chinese drum fed diets supplemented with *C*. *butyricum* at the dose of 1 x 10^8^ cells g^-1^ diet. After challenge with *Vibrio parahaemolyticus*, the cumulative mortality of Pacific white shrimp (*Litopenaeus vannamei*) significantly decreased with dietary *C*. *butyricum* at the dose of 1 x 10^8^ CFU g^-1^diet [19] and 1 x 10^5^, 1 x 10^7^, 1 x 10^8^, and 1 x 10^9^ CFU g^-1^ diet [20]. These results revealed that *C*. *butyricum* could improve the disease resistance of aquatic animals.

Disease resistance is often related to humoural immunity, and the complement system is an important part of humoral immunity [70]. In fish, C3 and C4 play important roles in combatting bacterial pathogens [58]. Studies have shown that the probiotics *Pediococcus acidilactici* [54,71], *Lactobacillus rhamnosus* [72], *Enterococcus faecium* [73,74] and *L*. *plantarum* [5] can improve serum complement component levels in fish. However, no studies have examined the effect of *C*. *butyricum* on serum complement in fish. In this study, serum C3 and C4 concentrations were improved in tilapias fed a diet supplemented with *C*. *butyricum* at levels of 1 x 10^5^, 1 x 10^6^, 1 x 10^7^CFU g^-1^ diet. Similar results were reported in which dietary *C*. *butyricum* improved serum C3 and C4 concentrations in broiler chickens [48,49]. The improvement of serum complement content induced by *C*. *butyricum* might be related to lipoteichoic acid (LTA). LTA is a major cell wall component of gram-positive bacteria [75,76]. In human, the classical complement pathway is activated by LTA *in vitro* [77]. In addition, immunity is related to antioxidative status in fish [78]. T-AOC is an anti-oxidative biomarker [79], and MDA is a typical parameter used to reflect oxidative injury [80]. In the present study, serum T-AOC was significantly increased and MDA content was significantly decreased in all four *C*. *butyricum* supplementary groups. Similar results were reported showing that dietary *C*. *butyricum* improved the antioxidant capacity in shrimp [20,41,42] and fish [66,81]. The effect of *C*. *butyricum* on antioxidant capacity of aquatic animals might be attributed to butyrate and H_2_. *C*. *butyricum* can produce butyric acid [82,83] and H_2_ [84]. Butyrate has been shown to reduce H_2_O_2_-induced DNA damage in human colon tumor cell [85] and to decrease MDA concentrations in the Caco-2 human colon carcinoma cell line [86]. Zhou et al. [87] reported that H_2_ reduced oxidative stress in acute pancreatitis both *in vitro* and *in vivo*.

The intestine is an important immune organ in fish [35]. Disease resistance in fish is related to intestinal immunity [25]. Cytokines play important roles in intestinal immunity [88]. Studies have shown that the gene expression of *TNF-α* and *IL-8* in the fish intestine is upregulated by the dietary probiotics *P*. *acidilactici* [12] and *L*. *plantarum* [27]. Our results showed that the gene expression of *TNF-α* and *IL-8* in the intestine was upregulated in tilapias fed a diet supplemented with *C*.*butyricum* at doses of 1 x 10^5^, 1 x 10^6^ and 1 x 10^7^ CFU g^-1^ diet. Similar trends were reported in HT-29 human colonic epithelial cells [17,30] and the jejunal mucosa of broiler chickens [39]. The effect of *C*. *butyricum* on cytokines might be related to butyric acid. Sodium butyrate was found to upregulate the gene expression levels of *TNF-α* in the intestine of juvenile common carp [89] and in bovine mammary epithelial cells [90]. IL-10 is an anti-inflammatory cytokine that suppresses excessive immune responses [33]. In the present study, the gene expression of *IL-10* was not influenced by dietary *C*. *butyricum*. This result differs from previous reports that *C*. *butyricum* upregulated the gene expression of *IL-10* in HT-29 human colonic epithelial cells [17,31]. This difference might be related to immune status. In mouse intestinal macrophages, *C*. *butyricum* failed to increase the production of IL-10 in the steady state, whereas it induced IL-10 production under inflamed conditions [33]. The TLR2/MyD88/IRAK-4 signalling pathway plays an important role in intestinal immune responses, inducing the production of cytokines such as TNF-α and IL-8, in mammals [91]. Study has shown that the probiotic *Psychrobacter sp*. SE6 upregulated gene expression of *TLR2* and *MyD88* in the intestine of grouper (*Epinephelus coioides*) [28]. There has been no investigation of the effect of TLR signalling pathways in immune responses induced by *C*. *butyricum* in aquatic animals. In the present study, *TLR2* gene expression was upregulated by dietary *C*. *butyricum* at levels of 1 x 10^6^ and 1 x 10^7^ CFU g^-1^ diet. *MyD88* gene expression was significantly upregulated by dietary *C*. *butyricum* at levels of 1 x 10^5^, 1 x 10^6^ and 1 x 10^7^ CFU g^-1^ diet. The gene expression of *IRAK-4* was significantly upregulated in all four *C*. *butyricum* supplementary groups. Similar results were obtained showing that *C*. *butyricum* upregulate *TLR2* gene expression in HT-29 human colonic epithelial cells [17], and upregulated the gene expression of *TLR2* and *MyD88* in the intestines of weaning rex rabbits [34]. The correlation analysis showed that *TNF-α* gene expression was significantly and positively related to gene expression of *TLR2* (r = +0.932, *P* < 0.05), *MyD88* (r = +0.969, *P* < 0.05) and *IRAK-4* (r = +0.960, *P* < 0.05) and that *IL-8* gene expression was significantly and positively related to gene expression of *TLR2* (r = +0.957, *P* < 0.05), *MyD88* (r = +0.936, *P* < 0.05) and *IRAK-4* (r = +0.936, *P* < 0.05). These results indicated that the upregulation of *TNF-α* and *IL-8* in response to dietary *C*. *butyricum* supplementation might be related to the upregulation of *TLR2*/*MyD88*/*IRAK-4* signalling molecules in tilapia. To the best of our knowledge, this is the first study of the effects of *C*. *butyricum* on *TLR2*/*MyD88*/*IRAK-4* signalling molecules in fish. The regulation of *TLR2*/*MyD88*/*IRAK-4* signalling molecules by *C*. *butyricum* might be related to LTA. Rashidi et al. [92] reported that the gene expression of *TLR2* and *MyD88* was upregulated by LTA in human whole endometrial cells.

On the other hand, the effect of *C*. *butyricum* on intestinal immunity may be related to the modulation of intestinal microbial components. Studies have shown that the diversity and composition of the intestinal microbiota affect immune responses, including the regulation of immune cells, cytokines and TLR signalling molecule [93–95]. It has been reported that probiotics can regulate intestinal communities in tilapia [29,96–98]. In the present study, we investigated intestinal microbial composition in the CB2 and CG groups. Our results showed that a total of 49 phyla were identified, the relative abundances of 42 phyla were significantly different between the CG and CB2 groups, and the phylum with the greatest change was Fusobacteria, followed by CKC4, Bacteroidetes and Firmicutes. *Cetobacterium* is present in high numbers in many fish species [99–102] and produces large quantities of vitamin B12 [103]. In the present study, *Cetobacterium* was the dominant bacteria of Fusobacteria in the two groups, and its relative abundance was significantly decreased in the CB2 group. Similar to our results, a previous study showed that *Cetobacterium* abundance was significantly decreased by a multi-species probiotic (from 13.8% to 0.02%) in tilapia [96]. The reason that *Cetobacterium* was inhibited by probiotics was unclear and needs further research. CKC4, a phylum in the SILVA database, was detected in the intestines of zebrafish [104], white shrimp (*Penaeus vannamei*) [105] and Chinese mitten crab (*Eriocheir sinensis*) [106]. In the present study, dietary *C*. *butyricum* significantly decreased the relative abundance of CKC4. However, there is a lack of detailed information about the function of CKC4 in the fish intestine. The specific mechanism needs further research. Firmicutes is an abundant phylum in the tilapia intestine [96] and provides a good index of intestinal health [47]. In the present study, Firmicutes was enriched in the CB2 group relative to the CG group. Meanwhile, the relative abundance of *Bacillus* was significantly improved in the CB2 group. These results suggested that dietary *C*. *butyricum* could stimulate the growth of beneficial Firmicutes bacteria, such as *Bacillus*, in the tilapia intestine. A similar result was reported showing that Firmicutes, such as *Bacillus* and *Clostridium*, were abundant in Pacific white shrimp (*Litopenaeus vanname*i) fed a diet supplemented with *C*. *butyricum* [47]. Various opportunistic pathogens cause disease in stressed fish and fish affected by concurrent infection [107]. *Aeromonas hydrophila* is a conditional pathogen [108]. In the present study, the relative abundance of *Aeromonas* was significantly decreased in the CB2 group. A similar result was reported in Pacific white shrimp (*Litopenaeus vannamei*), showing that dietary *C*. *butyricum* inhibited some intestinal opportunistic pathogen genera [47]. In vitro, *C*. *butyricum* exhibited significant inhibitory activity on the growth and adherence of pathogenic bacteria to fish intestinal epithelial cells [45,46]. These results revealed that *C*. *butyricum* might decrease the risk of opportunistic pathogens to invade the host. Compared with disease states, there was a higher microbial diversity index in healthy humans [109], mice [110], pigs [111] and fish [110]. Stress caused a reduction in intestinal microbial diversity in fish [97]. In this study, alpha-diversity indices, including Sobs, Shannon, ACE and Chao1, were significantly increased by dietary *C*. *butyricum*. Similarly, *C*. *butyricum* increased the alpha-diversity indices, including ACE and Chao1, in Pacific white shrimp (*Litopenaeus vannamei*) [47]. These results indicated that *C*. *butyricum* improved the homeostasis of the intestinal microbial community.

In fish, the intestinal immunity is correlated with intestinal structural integrity [35]. DAO is an index of intestinal mucosal cell integrity [39]. In this study, serum DAO activity was decreased in tilapia that were fed diets supplemented with *C*. *butyricum* at all four levels. Similar results were reported in broiler chickens [39] and weaned piglets [40]. These results indicate that *C*.*butyricum* can improve intestinal mucosal cell integrity in fish. The observed decrease of DAO activity in the serum of fish fed *C*. *butyricum* might be associated with butyric acid. Butyric acid is a major respiratory fuel, is trophic to the colon and exerts proliferative effects on colonocytes [112]. Fang et al. [113] reported that serum DAO was significantly decreased by sodium butyrate in weanling piglets. In previous work, the intestinal intercellular structural integrity was found to be related to tight junction proteins, such as occludin and claudin, in the Caco-2 human colon carcinoma cell line [114]. Dietary *C*. *Butyricum* upregulated the protein expression levels of *ZO-1*, *claudin-3*, *occludin* in intestine of weaned piglets [40] and the gene expression levels of *ZO-1* and *occludin* in ileum and colon of weaning rex rabbits [34]. In the present study, *C*. *butyricum* had no effect on the gene expression of *claudin-1* and *occludin* in tilapia intestine. Nébot-Vivinus et al. [115] reported that in the T84 human colon epithelial cell line, multispecies probiotic LT did not affect the expression of *occludin* under basal conditions, but the expression of *occludin* was increased upon lipopolysaccharide stimulation. The effects of probiotics on tight junction protein might be related to health.

## Conclusions

Dietary *C*. *butyricum* at a suitable dose significantly improved the growth performance, humoural and intestinal immunity, and integrity of the intestinal structure, elevated the diversity of the intestinal microbiota and the relative abundance of beneficial bacteria, decreased the relative abundance of opportunistic pathogenic bacteria, and enhanced the disease resistance in tilapia. According to the growth performance, immunity and disease resistance, the optimal dose of dietary *C*. *butyricum* was 1 x 10^5^ CFU g^-1^ diet.
